# Pharmacokinetics and Bioequivalence of Isavuconazole Administered as Isavuconazonium Sulfate Intravenous Solution via Nasogastric Tube or Orally in Healthy Subjects

**DOI:** 10.1128/AAC.00442-21

**Published:** 2021-08-17

**Authors:** Amit Desai, Melanie Helmick, Nakyo Heo, Selina Moy, Stephen Stanhope, Ronald Goldwater, Nancy Martin

**Affiliations:** a Astellas Pharma Global Development, Northbrook, Illinois, USA; b Parexel International Corporation, Baltimore, Maryland, USA

**Keywords:** isavuconazole, nasogastric tube, bioequivalence

## Abstract

For critically ill patients with invasive fungal infections, a nasogastric (NG) tube can be an alternative route for administration of isavuconazonium sulfate (ISAVUSULF). This was a randomized, open-label, 2-period, 2-sequence single-dose crossover study comparing single doses of 372 mg ISAVUSULF intravenous (i.v.) solution via NG tube (test formulation) to 372-mg ISAVUSULF capsules for oral administration (reference formulation) in healthy male and female subjects. A single dose of ISAVUSULF was administered under fasting conditions on day 1 of each period, with a washout of 30 days between periods. Pharmacokinetic (PK) samples were collected predose through day 21. Standard safety and tolerability assessments were conducted in each period. The analysis of variance estimate of the study population demonstrates that the isavuconazole i.v. NG tube administration geometric least-squares (LS) mean values of the observed maximum concentration (*C*_max_), area under the plasma concentration-time curve (AUC) to the last measurable concentration (AUC_last_), AUC to time infinity (AUC_inf_), and AUC from start of dosing to 72 h (AUC_72_) were 105.3%, 97.6%, 99.3%, and 97.8%, respectively, of the corresponding oral-administration values. The geometric LS mean ratio and 90% confidence intervals for the PK parameters were completely contained within the prespecified limits of 80% to 125%. There were no deaths or serious adverse events that led to the withdrawal of treatment during the study. The study met its primary endpoint of bioequivalence between the two routes of administration. Both routes of administration were well tolerated.

## INTRODUCTION

Isavuconazonium sulfate (ISAVUSULF) is the water-soluble prodrug of the active triazole isavuconazole (ISA), an antifungal agent that inhibits sterol 14α-demethylase, a microsomal P450 enzyme essential for ergosterol biosynthesis in fungi (1). ISAVUSULF has been approved as a broad-spectrum antifungal agent for the treatment of invasive aspergillosis and invasive mucormycosis in adults. It is a well-tolerated and easily administered antifungal agent and therefore addresses a substantial medical need for the treatment of invasive fungal infections in response to the emergence of fungal isolates resistant to existing azoles, the limited spectrum of the echinocandins, and the toxicity of amphotericin B (2).

ISAVUSULF has both intravenous (i.v.) and oral formulations. The pharmacokinetics (PK) of ISA are linear (3, 4), with minor intra- and intersubject variability in healthy subjects (Astellas Pharma, Inc., data on file; 5), an oral bioavailability of >98%, and no relevant food effects (5). However, there are very limited data regarding alternative routes of administration for ISAVUSULF and its PK properties (6).

Nasogastric (NG) tube feeding is most commonly used in the intensive care unit. It is also used in patients who are unable to eat, for example, cancer patients with mucositis or patients with severe nausea who do not want to eat (7). As there is an unmet medical need in patients for whom oral administration is impractical or in whom an i.v. port is not available, administration of ISAVUSULF i.v. solution via NG tube may represent an alternate route of oral administration.

The primary objective of this study was to assess the bioequivalence of ISAVUSULF i.v. solution via NG tube (test formulation) versus ISAVUSULF oral capsules (reference formulation). The secondary objective was to monitor the safety and tolerability of two single doses of ISAVUSULF.

## RESULTS

### Patients.

Eighteen healthy subjects (Table 1) were randomized in this study, of whom 10 (55.6%) were male and eight (44.4%) were female. The mean age was 37.1 years (range, 24 to 52 years). The population was primarily Black or African American (14 [77.8%] subjects). Thirteen subjects provided plasma concentrations in both periods that were pharmacokinetically evaluable.

**TABLE 1:** Demographics and characteristics of the study group

Parameter and statistic^*a*^	Value for patient group^*b*^		
	AB	BA	Total (*n* = 18)
Sex [no. (%)]			
Male	6 (60.0)	4 (50.0)	10 (55.6)
Female	4 (40.0)	4 (50.0)	8 (44.4)
Ethnicity [no. (%)]			
Hispanic or Latino	1 (10.0)	0	1 (5.6)
Not Hispanic or Latino	9 (90.0)	8 (100.0)	17 (94.4)
Race [no. (%)]			
White	2 (20.0)	2 (25.0)	4 (22.2)
Black or African American	8 (80.0)	6 (75.0)	14 (77.8)
Asian	0	0	0
Native Hawaiian or other Pacific Islander	0	0	0
Other	0	0	0
Age (yrs)			
Mean (SD)	36.0 (5.9)	38.5 (11.3)	37.1 (8.5)
Median	36.5	38.0	36.5
Min–max	28–43	24–52	24–52
EudraCT age category [no. (%)]			
≥18 yrs to ≤64 yrs	10 (100)	8 (100)	18 (100)
Wt (kg)			
Mean (SD)	83.47 (15.71)	83.11 (10.01)	83.31 (13.11)
Median	80.75	83.55	82.10
Min–max	57.6–109.2	70.0–97.4	57.6–109.2
Ht (cm)			
Mean (SD)	174.7 (9.4)	169.6 (10.4)	172.4 (9.9)
Median	176.0	171.0	175.0
Min–max	160–188	157–182	157–188
BMI (kg/m^2^)			
Mean (SD)	27.11 (2.96)	28.88 (2.09)	27.89 (2.69)
Median	26.35	29.25	28.85
Min–max	22.5–31.3	24.7–31.3	22.5–31.3

a BMI, body mass index; max, maximum; min, minimum; SD, standard deviation; EudraCT, European Union Drug Regulating Authorities Clinical Trials Database.

b AB and BA indicate treatment sequence, as follows: A, 372 mg isavuconazonium sulfate intravenous solution via nasogastric tube (test formulation); B, 372-mg (two 186-mg capsules) isavuconazonium sulfate oral capsules (reference formulation).

### Pharmacokinetics.

**FIG 1:**
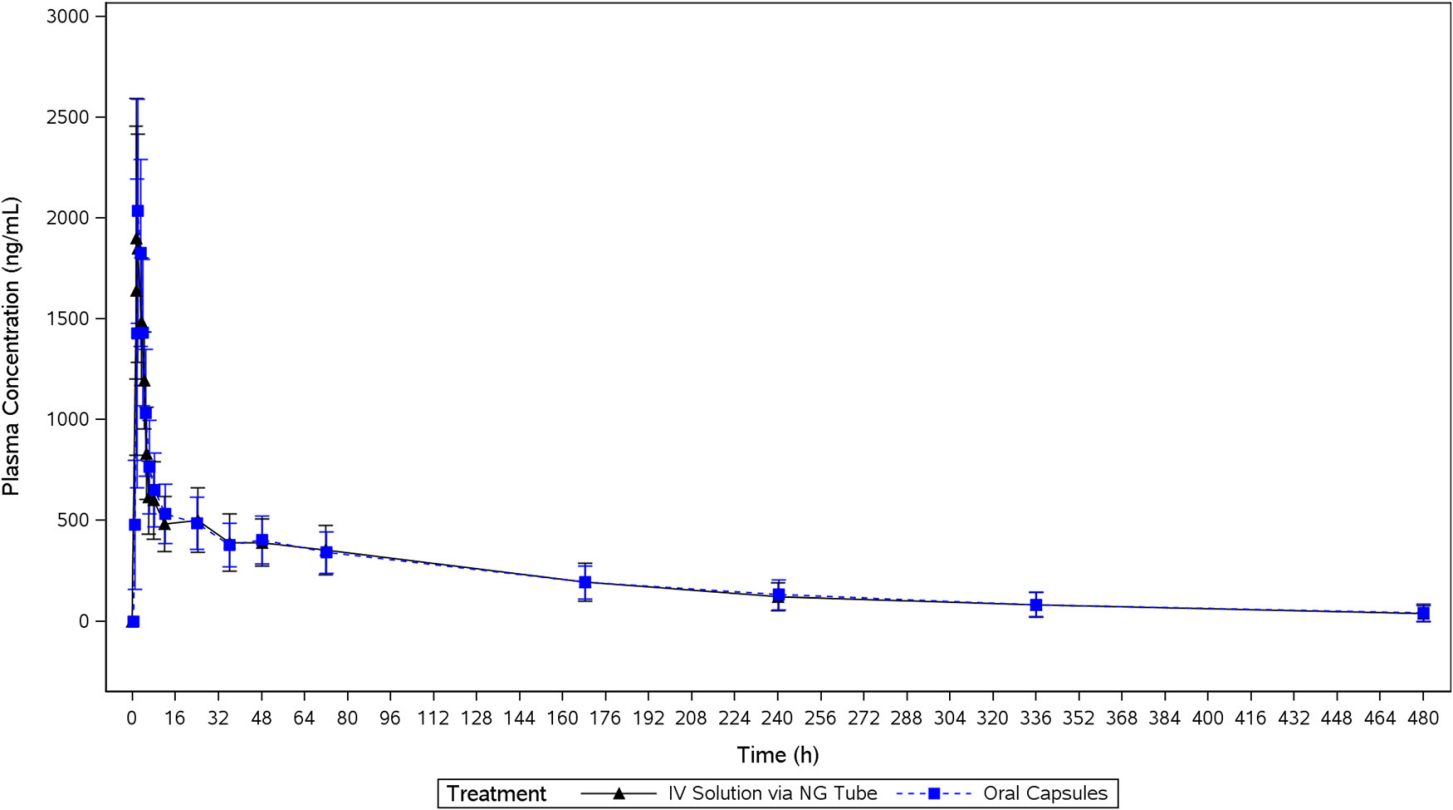
Mean (SD) plasma concentration of isavuconazole by method of treatment administration (linear scale plot) (pharmacokinetic analysis set). IV, intravenous; NG, nasogastric tube.

**FIG 2:**
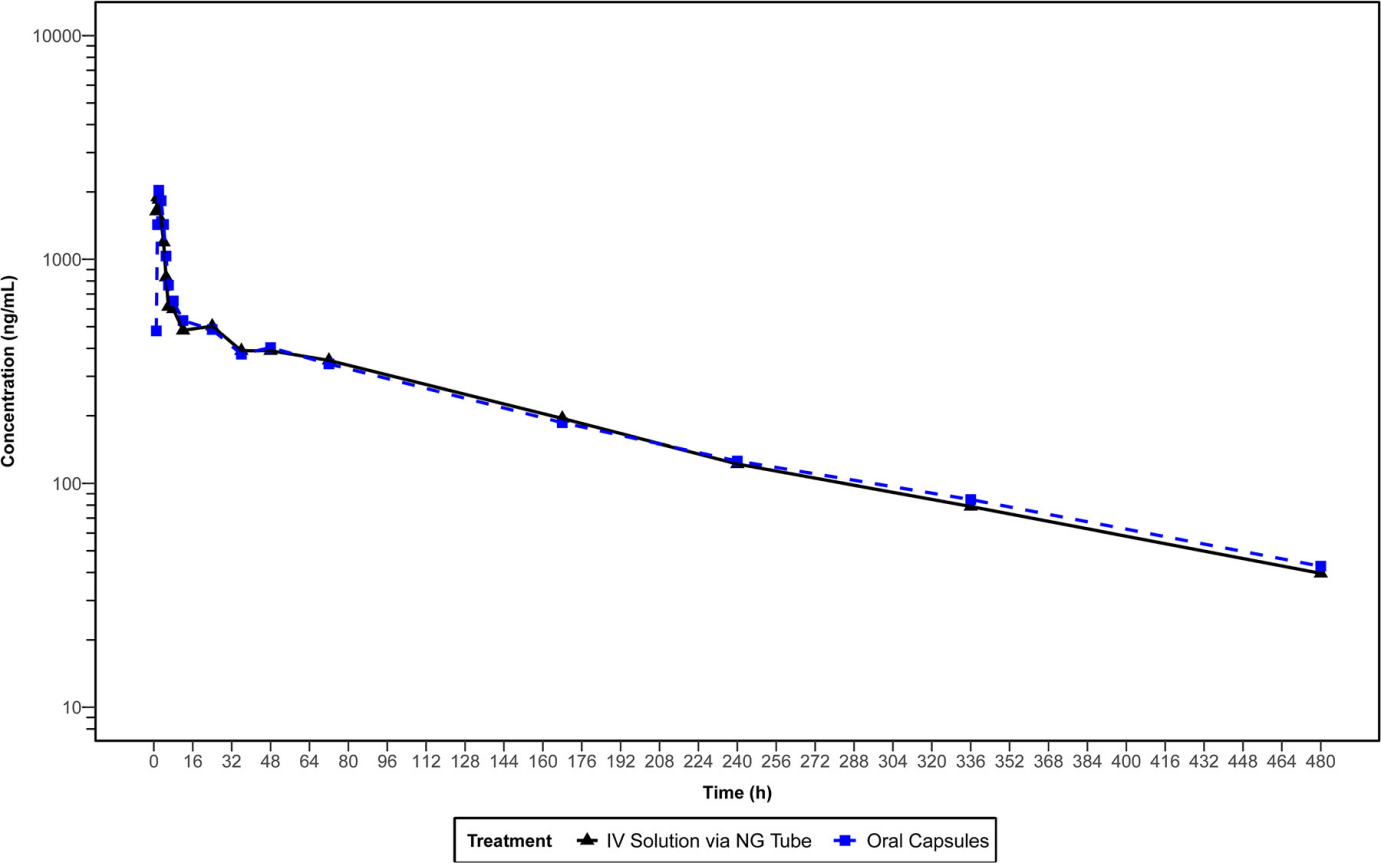
Mean (SD) plasma concentration of isavuconazole by method of treatment administration (semilog plot) (pharmacokinetic analysis set).

**FIG 3:**
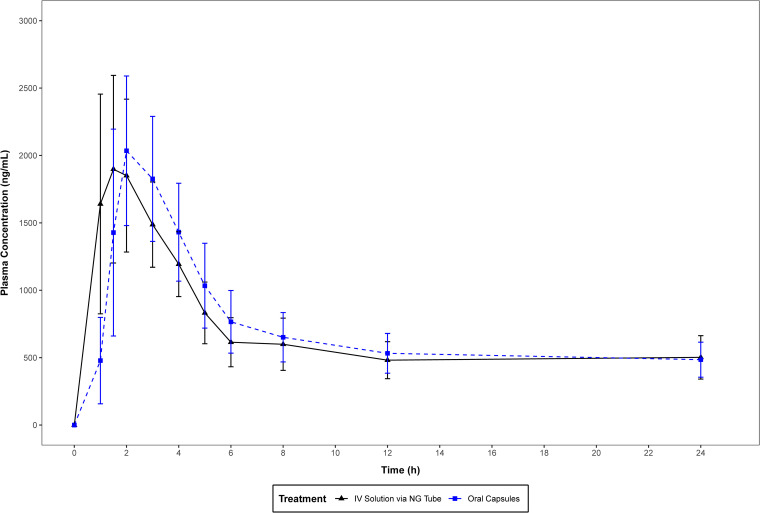
Mean (SD) plasma concentration (0 to 24 h) of isavuconazole by method of treatment administration (linear scale plot) (pharmacokinetic analysis set).

The mean plasma concentration-time profiles of ISA administered as ISAVUSULF by formulation on a linear and semilog scale are presented in Fig. 1 and 2, respectively. The mean plasma concentration plot of ISA truncated to the first 24 h is presented in Fig. 3. The mean concentration-time profiles of both formulations, i.v. solution via NG tube and oral capsules, were similar in shape. Each measured PK parameter was similar for subjects in both formulations (Tables 2 and 3). All mean parameters were similar between the two formulations.

**TABLE 2:** Summary of primary pharmacokinetic parameters

Parameter and statistic^*a*^	Value for ISAVUSULF treatment group	
	Intravenous solution via NG tube (test formulation)	Oral capsules (reference formulation)
AUC_inf_ (h·ng/ml)		
No.	12	12
Mean (SD)	98,142 (43,628)	100,050 (46,826)
% CV	44.5	46.8
AUC_72_ (h·ng/ml)		
No.	13	13
Mean (SD)	34,885 (7,708)	35,777 (8,809)
% CV	22.1	24.6
AUC_last_ (h·ng/ml)		
No.	13	13
Mean (SD)	85,869 (28,873)	88,492 (31,722)
% CV	33.6	35.8
*C*_max_ (ng/ml)		
No.	13	13
Mean (SD)	2,295 (542)	2,185 (582)
% CV	23.6	26.7

a CV, coefficient of variation; SD, standard deviation.

**TABLE 3:** Summary of secondary pharmacokinetic parameters

Parameter and statistic^*a*^	Value for ISAVUSULF treatment group	
	Intravenous solution via NG tube (test formulation)	Oral capsules (reference formulation)
CL/*F* (liters/h)		
No.	13	12
Mean (SD)	2.38 (0.961)	2.41 (1.05)
% CV	40.4	43.9
*t*_1/2_ (h)		
No.	13	12
Mean (SD)	132 (69.1)	121 (64.5)
% CV	52.3	53.2
*t*_lag_ (h)		
No.	13	13
Median	0	0
*t*_max_ (h)		
No.	13	13
Median	1.50	2.00
*V*_z_/*F* (liters)		
No.	13	12
Mean (SD)	390 (129)	356 (100)
% CV	33.1	28.3

a CV, coefficient of variation; SD, standard deviation.

Results of the statistical analysis assessment of the bioequivalence of the primary parameters of ISA between ISAVUSULF i.v. solution via NG tube (test formulation) compared to ISAVUSULF oral capsules (reference formulation) are presented in Table 4. The geometric least-squares (LS) mean values for the observed maximum concentration (*C*_max_), area under the plasma concentration-time curve (AUC) to the last measurable concentration (AUC_last_), AUC to time infinity (AUC_inf_), and AUC from start of dosing to 72 h (AUC_72_) for ISAVUSULF i.v. solution via NG tube were 105.3%, 97.6%, 99.3%, and 97.8% of the corresponding values for oral capsules, respectively. The geometric LS mean ratios and their 90% confidence intervals for *C*_max_, AUC_last_, AUC_inf_, and AUC_72_ were completely contained within the prespecified limits of 80% to 125%.

**TABLE 4:** Statistical assessment of the bioequivalence of isavuconazonium sulfate administered by intravenous solution via nasogastric tube compared to oral capsules (pharmacokinetic analysis set)

Parameter	ISAVUSULF i.v. solution via NG tube (test formulation)		ISAVUSULF oral capsules (reference formulation)		Geometric LS mean ratio (%)^*a*^	90% CI of ratio^*a*^
	*n*	Geometric LS mean	*n*	Geometric LS mean		
AUC_inf_ (h·ng/ml)	12	90,300	12	91,000	99.27	93–106
AUC_72_ (h·ng/ml)	13	34,000	13	34,700	97.81	93–103
AUC_last_ (h·ng/ml)	13	81,400	13	83,400	97.60	92–103
*C*_max_ (ng/ml)	13	2,230	13	2,120	105.34	89–124

a Ratios and confidence limits are back-transformed to the raw scale, and values are expressed as percentages.

### Safety.

Overall, 11 treatment-emergent adverse events (TEAEs) were reported for three (17.6%) subjects during the study. One (5.9%) of these subjects experienced single episodes of procedural nausea and procedural vomiting due to the NG tube, which led to withdrawal of treatment. All TEAEs were considered by the investigator to be mild in severity, except for upper respiratory tract infection (reported for one subject) and headache (reported for one subject). These were considered by the investigator to be moderate in severity. No TEAEs were considered by the investigator to be drug related, and no serious AEs led to the withdrawal of treatment during the study. There were no deaths during the study. No potentially clinically significant vital signs measurements were reported as AEs across sequences. Electrocardiogram (ECG) abnormalities (sinus bradycardia, early repolarization normal variant, normal sinus rhythm with sinus arrhythmia, first degree atrioventricular block) were observed across both sequences; however, none of these abnormalities was considered clinically significant.

## DISCUSSION

In this study, the pharmacokinetics of ISAVUSULF i.v. solution via NG tube and ISAVUSULF oral capsules were compared to assess the bioequivalence of the two formulations. Data from healthy subjects showed that NG tube administration of an i.v. solution of ISAVUSULF resulted in *C*_max_, AUC_last_, AUC_inf_, and AUC_72_ PK parameters similar to those obtained with ISAVUSULF oral capsules. Geometric LS mean ratios and 90% confidence intervals (CIs) were completely contained within the prespecified limits of 80% to 125%, indicating bioequivalence between the two routes of administration. Parameter values from this study are comparable to those previously reported for healthy subjects (4). Parameter values are also comparable to those seen with i.v. and oral treatment in phase 3 clinical trial in patients with infections caused by Aspergillus and other filamentous fungi (8). Our results indicate that ISAVUSULF i.v. solution can be administered via NG tube and provide an alternative route of administration for critically ill patients who cannot swallow capsules or for patients where i.v. administration is not possible.

Administration of ISAVUSULF via other routes of administration has recently been reported. A recent study by McCreary et al. investigated ISAVUSULF for the prevention and treatment of invasive fungal infection in transplant recipients (6). ISAVUSULF capsules were opened and their contents sprinkled into an enteral feeding tube (6). The team found that approximately 90% of ISA trough concentrations attained in their study were comparable to previously reported trough concentrations in patients (8, 9). These concentrations were also comparable to data from real-world ISA samples, in which approximately 90% of reported concentration values were >1,000 ng/ml (10). In a case report by Adamsick et al., ISAVUSULF capsules were opened and a saline slurry was produced for administration into the gastro-jejunum tube, in a patient with a complex post‐lung transplant course for presumed invasive aspergillosis (11). The trough concentrations reported in this study were similar to previously reported concentrations in patients (8, 9). However, it is important to note that opening the ISAVUSULF capsule is not recommended, as the drug substance is hygroscopic (6). Administering ISAVUSULF from an open capsule could therefore introduce variability in concentration measurements, depending on the handling of the open capsules.

Data have also been published for other azoles administered via either NG tube or other feeding tubes. In a phase 1, open-label, single-center, randomized, crossover study, posaconazole was administered via NG tube or orally (12). The LS mean values for *C*_max_, AUC_last_, and AUC_inf_ for posaconazole administered via NG tube compared to oral administration were 81%, 76%, and 77%, respectively. The 90% CIs for *C*_max_, AUC_last_, and AUC_inf_ were all outside the 80%-to-125% range (12). Itraconazole was administered to a patient with cystic fibrosis by NG tube, in which beads of the drug were dissolved in 15 to 30 ml of cranberry juice (13). Measured concentrations were within its therapeutic range when itraconazole was administered by NG tube. Voriconazole was administered to a patient with infection due to Candida glabrata by jejunostomy tube. In this single-patient case study, blood drug levels of voriconazole delivered as crushed tablets via a jejunostomy tube were similar to those achieved via oral administration (14). In a prospective observational study, eight adult subjects with suspected or documented invasive fungal infection were administered a suspension of crushed voriconazole tablets via NG tube (15). Of eight patients, one patient had lower *C*_max_ and trough concentrations. There was no clear reason or explanation for the lower concentrations (15). Overall, results of administration of ISAVUSULF via NG tube were comparable to results with other azoles, except for posaconazole, when administered via either NG or feeding tube as described above. However, there were differences in study design for other azoles, including notable differences in the formulation and number of subjects administered drug via NG tube. In some cases, data were available from a single-subject case study instead of a well-designed study.

ISAVUSULF administered via NG tube was well tolerated, with no TEAEs considered by the investigator to be drug related. There was only one case of treatment withdrawal, which was due to mild TEAEs. No subjects demonstrated clinically significant laboratory values for hepatoxicity or had concomitant elevations in alanine transaminase or aspartate transaminase that required further liver function investigation.

The current study was conducted in healthy subjects. Administration of ISAVUSULF via NG tube is not tested in critically ill patients. Absorption of ISA via NG tube in critically ill patients (or those who are not critically ill but are not young, healthy adults) is unknown.

### Conclusion.

These data demonstrate the bioequivalence of ISAVUSULF i.v. solution via NG tube and ISAVUSULF oral capsules. Both routes of administration were considered safe and well tolerated in healthy male and female subjects.

## MATERIALS AND METHODS

### Inclusion/exclusion criteria.

Eligible participants were either male or female healthy subjects aged 18 to 55 years, with a body mass index of 18.5 to 32.0 kg/m^2^. Female subjects were required to be of non-childbearing potential or to have negative pregnancy test results at screening. Breastfeeding was not permitted at screening, throughout the study period, and for 30 days after final study drug administration. In addition, male subjects with female partners of childbearing potential (including breastfeeding partners) had to have agreed to remain abstinent or use a condom for the duration of the pregnancy, throughout the study period, and for 30 days after final drug administration.

Subjects were excluded from the study if they had received any investigational therapy within 28 days or 5 half-lives, whichever was longer, prior to screening. Subjects were also excluded if they had any condition which, in the investigator’s opinion, made the subject unsuitable for study participation; had been pregnant or breastfeeding within 6 or 3 months, respectively, prior to screening; or had a history of peptic or gastric ulcers. In addition, subjects were excluded if they had a history of sinus disease, sinus allergy, rhinoplasty or any surgery of the nose, septum, or nasal passages, or any other abnormality that, in the opinion of the investigator, could have impacted NG tube placement (e.g., nasal polyps). Subjects were also excluded if they had taken medication or substances via inhalation through the nasal passages within 3 months prior to screening.

This study was conducted in accordance with the ethical principles that have their origin in the Declaration of Helsinki, Good Clinical Practice (GCP), International Council for Harmonization of Technical Requirements for Pharmaceuticals for Human Use (ICH) guidelines, and applicable laws and regulations. An institutional review board-approved written informed consent was obtained from each subject prior to the initiation of any study-specific procedures.

### Study design.

This was a randomized, open-label, 2-period, 2-sequence single dose crossover study in healthy male and female subjects. ISA has an intrasubject coefficient of variation (CV) of 5% for AUC and 12% for *C*_max_ (Astellas Pharma, Inc., data on file; 5). Assuming treatment ratios to be 100% for AUC and *C*_max_, a sample size of 6 subjects in each sequence provided an overall power of at least 90% to demonstrate bioequivalence between the test and reference ISAVUSULF formulations, with the 90% CI for the true underlying ratio of AUC and *C*_max_ within the range of 80.00% to 125.00%. An additional 2 subjects, 1 within each sequence, were enrolled to compensate for potential dropouts. Healthy subjects were randomized in a 1:1 ratio to receive either a single dose of 372 mg ISAVUSULF i.v. solution via NG tube (test formulation) followed by a single oral dose of 372 mg (two 186-mg capsules) ISAVUSULF capsules (reference formulation), or vice versa. Subjects received a single dose of ISAVUSULF (either formulation) under fasting conditions (i.e., no food or beverage was allowed from at least 10 h predose through 4 h postdose) on day 1 of each treatment period. Treatment periods were separated by a washout of at least 30 days between study drug administration in each period (more than five times the 130-h elimination half-life) (4). Water intake was prohibited from 1 h predose through 1 h postdose, except for 240 ml water to swallow the drug in the case of oral administration. For NG tube administration, one vial of ISAVUSULF was reconstituted by adding water (5 ml). The entire contents of the vial were administered as a solution via NG tube, followed by three 5-ml rinses with water. The reconstituted solution was stored below 25°C for a maximum of 1 h before administration. Correct placement of the NG tube was confirmed using X-ray radiography, and then the dose was delivered through the NG tube. Subjects were screened up to 28 days prior to study drug administration on day 1, period 1. Eligible subjects were admitted to the clinical unit on day −1 and were confined to the study center for 5 days. Subjects returned to the study center on days 8, 11, 15, and 21 to collect pharmacokinetic samples.

### Pharmacokinetics assessment.

Blood samples were collected predose on day 1 and at the following postdose time points in each period: 1, 1.5, 2, 3, 4, 5, 6, 8, 12, 24, 36, 48, 72, 168, 240, 336, and 480 h. The following primary pharmacokinetic parameters were assessed: *C*_max_, AUC_last_, AUC_inf_, and AUC_72_. Secondary parameters assessed were apparent clearance (CL/*F*), terminal elimination half-life (*t*_1/2_), time of maximum concentration (*t*_max_), lag time (*t*_lag_), and apparent volume of distribution (*V*_z_/*F*) during the terminal phase. Individual ISA plasma concentrations were used to estimate the derived pharmacokinetic parameters using model-independent methods. The linear up-log down trapezoidal method was used to calculate the PK parameters in Phoenix WinNonlin (Certara, St. Louis, MO). Summary statistics were calculated for the pharmacokinetic parameters.

### Bioanalytical methods.

ISA concentrations in plasma samples were measured using a validated liquid chromatography-tandem mass spectrometry (LC-MS/MS) method at Pharmaceutical Product Development, LLC (PPD, Middleton, WI). A plasma sample (50 μl) was combined with the internal standard (*d*_4_-ISA/pyridooxazinone) and subjected to protein precipitation using acetonitrile. Following centrifugation for 10 min at 5°C, supernatant (75 μl) was isolated for dilution with a solution of water-formic acid-ammonium hydroxide (1,000:10:0.5 [vol/vol/vol])–acetonitrile (850:150 [vol/vol]) and analyzed. The calibration curve was constructed using peak area ratios of the calibration standards by applying a linear, 1/concentration-weighted, LS regression algorithm. The range of the calibration curve was 100 ng/ml to 10,000 ng/ml with the lower limit of quantification set to 100 ng/ml. The quality control samples were prepared at concentrations of 200, 4,000, and 8,000 ng/ml. The intrasubject accuracy and precision were between −3.22 and 4.75% relative error (RE) and <5.80% relative standard deviation (RSD). The intersubject accuracy and precision were between −0.243 and 2.40% RE and <3.55% RSD. Three freeze-thaw cycles were validated. No dilutions were applied during the analysis.

A subset of samples measuring <100 ng/ml postdose were reassayed, with a calibration curve of 5.00 to 1,250 ng/ml and a lower limit of quantification of 5.00 ng/ml. The quality control samples were prepared at concentrations of 12.5, 500, and 1,000 ng/ml. The intrasubject accuracy and precision were between −9.60 and 2.50% RE and <2.48% RSD. The intersubject accuracy and precision were between −5.64 and 1.10% RE and < 5.17% RSD. Five freeze-thaw cycles were validated, and no dilutions were applied during the analysis.

### Safety assessment.

Clinical laboratory tests included blood collection for serology tests (at screening only), hematology, and biochemistry. Samples were collected at screening and in each period on day −1 and day 2. Samples collected on day 2 were for liver function tests only. Blood pregnancy tests were conducted at screening. Vital signs included measurements of blood pressure and pulse, and these were taken at screening, in each period, and at the end of study visits. In each period, measurements were taken on day −1, predose on day 1, at 2, 8, and 24 h and day 4 postdose. A routine 12-lead ECG was taken at screening only, after the subject had been resting in the supine position for at least 5 min.

### Statistical analysis.

To evaluate the bioequivalence of the ISAVUSULF i.v. solution via NG tube (test formulation) compared to ISAVUSULF oral capsules (reference formulation), an analysis of variance (ANOVA) model with period and formulation as fixed effects, and subject as a random effect, was fitted on natural logarithmic-transformed values for AUC_inf_, AUC_last_, AUC_72_, and *C*_max_. Within the ANOVA, the LS mean differences and their corresponding 90% CIs for ISAVUSULF i.v. solution via NG tube and ISAVUSULF oral capsules were estimated. The geometric LS mean ratios and their corresponding 90% CIs for each pharmacokinetic parameter were also back-transformed and were expressed as percentages. The test formulation was considered bioequivalent to the reference formulation if the 90% CIs for the geometric LS mean ratios of each of the pharmacokinetic parameters were within the range of 80.00% to 125.00%.

### Data availability.

Researchers may request access to anonymized participant level data, trial level data, and protocols from Astellas sponsored clinical trials at www.clinicalstudydatarequest.com. The Astellas criteria for data sharing are available at https://clinicalstudydatarequest.com/Study-Sponsors/Study-Sponsors-Astellas.aspx.
